# Increased meaningful activity while social distancing dampens affectivity; mere busyness heightens it: Implications for well-being during COVID-19

**DOI:** 10.1371/journal.pone.0244631

**Published:** 2020-12-31

**Authors:** Daniel B. Cohen, Morgan Luck, Atousa Hormozaki, Lauren L. Saling

**Affiliations:** 1 School of Humanities and Social Sciences, Charles Sturt University, Wagga Wagga, New South Wales, Australia; 2 School of Health and Biomedical Sciences, RMIT University, Bundoora, Victoria, Australia; Koc University School of Medicine, TURKEY

## Abstract

Social distancing measures have been implemented in many countries to limit the spread of COVID-19. Emerging literature reveals that fear of acquiring COVID-19 has detrimental psychological ramifications. However, it seems likely that social distancing will have a further negative impact on well-being. The focus of this study was therefore to investigate whether changes in behaviour as a result of social distancing would predict changes in well-being. Participants (n = 95) rated their level of well-being as it was both during social distancing and retrospectively one month before beginning social distancing. Participants also indicated how much time they spent engaged in various activities both during social distancing and one month before social distancing and nominated how important each of these activities was for them. These measures employed scales created specifically for the present study. In addition, participants completed the Big Five Inventory–2 Extra-Short Form and the nine-item version of the Personal Optimism and Self-Efficacy Optimism Scale. We found that affectivity–both positive and negative–decreased with increased engagement in meaningful activities and that affectivity increased with increased activity in general. While both sorts of activity appear to improve some aspects of well-being, it appears that meaningful activity regulates psychological homeostasis while busyness in general does not.

## Introduction

COVID-19, caused by the novel coronavirus SARS-CoV-2, has achieved pandemic status. Many countries have instituted some form of social distancing to curtail its spread. Measures include the closure of schools, workplaces, cafes, and public venues, limits on the number of visitors allowed in private homes, and, in some cases, the restriction of people to their homes or to ‘quarantine hotels’ (with possible exceptions for exercise or to acquire essential items). While it is clear that COVID-19 has significant physical impacts including, in the most severe case, death, it is likely to have extensive psychological impacts as well. For instance, the general population is experiencing significant anxiety, depression and stress [1–6] as are healthcare workers who manage patients with COVID-19 [7,8]. These psychological impacts result from three distinct aspects of the pandemic: (i) contracting the virus, (ii) fear of contracting the virus, and (iii) social distancing. We are specifically interested in isolating the effects of this third factor.

A number of studies investigating the psychological impact of social distancing are synchronic: that is, they measure well-being at just one time point. For instance, a sample of Canadian participants reported significant psychological distress associated with COVID-19 [9], while in a U.S. sample, health anxiety, financial worry, and loneliness were associated with being under stay-at-home orders due to COVID-19 [10]. However, synchronic studies provide only limited evidence concerning the effect of social distancing on well-being.

Better studies are diachronic: that is, they measure the well-being of a sample at a number of time points. For instance, a Dutch study [11] found that two months after social distancing measures were implemented, levels of emotional loneliness amongst older adults were significantly higher than levels reported in late 2019 with no other changes in well-being noted. A U.S. study [12] investigated loneliness immediately before the COVID-19 outbreak (baseline), during the first wave of social distancing (time 1), and during the second wave of social distancing (time 2). No change in loneliness over the three time points was found, aside from a slight increase in loneliness in adults aged ≥ 65 years from time 1 to time 2. In another U.S. study [13], social distancing was associated with significantly higher levels of depression, anxiety, and insomnia, compared to levels measured before social distancing. Finally, in a German study [14], women reported a decrease in family and work satisfaction during lockdown compared to pre-lockdown levels.

A limitation of extant diachronic studies is their focus on narrow aspects of well-being; while these factors may be indicative of the psychological impact of social distancing, they are not definitive. These studies also do not assess positive and negative affect separately. Although these two aspects are related, they can come apart.

The present study is diachronic: participants reported on their psychological well-being and activity both before and during social distancing. In contrast with other diachronic studies, we also conducted a broad measure of psychological well-being as opposed to focussing on particular dimensions such as loneliness. We also took well-being to be composed of two independent variables: positive and negative affect. This allowed us, in our analysis, to identify subtle implications for well-being that might otherwise be overlooked (in particular, factors that increase positive affect without decreasing negative affect, and vice versa).

A further limitation of extant studies is their failure to explore factors which might explain the psychological impact of social distancing. Given that social distancing affects the kinds of activities that one can engage in as well as impacting on how activities are typically undertaken, it is likely that this accounts for some of the psychological impact. Meaningful activity is positively associated with psychological well-being–specifically, life satisfaction and purpose in life [15]. Similarly, decreasing meaningful activity is associated with a decrease in well-being. For instance, in patients suffering from depression, a withdrawal from valued activities exacerbates depressive symptoms and, correspondingly, increasing meaningful behaviour reduces depressive symptoms [16]. Giving up valued activities due to chronic illness is associated with a decrease in well-being, while replacing these activities with other valued activities is associated with improvements in well-being [17]. Fewer depressive symptoms are reported by retirees when they engage in meaningful activities [18]. The literature suggests, therefore, that specific benefits are conferred by particular activities rather than by activity in general. In the current study we thus considered the psychological impact both of overall changes in activity, and of changes in meaningful activity, in particular, in the context of social distancing.

Research into well-being during a crisis tends to focus on stable individual differences as predictors of psychological well-being. For instance, high perceived control was found to protect against chronic stress in the aftermath of the Three Mile Island nuclear accident [19]. Similarly, optimism tends to promote resilience and self-efficacy in healthcare workers during disasters [20]. Although interesting, these variables are not readily modifiable. In the present study, we measured stable individual differences, but our focus was to investigate whether changes in behaviour during social distancing impact on well-being. This was the main aim of our study.

If a change in behaviour predicts a change in well-being, then, as behaviour is readily modifiable, this suggests a productive way to manage well-being during social distancing and related events which impact on activity, such as illness and disasters. We hypothesized that two factors would predict a change in well-being (measured by comparing well-being levels before social distancing with well-being levels during social distancing): (a) a change in total time spent on activities, and (b) a change in time spent on activities rated as meaningful by participants (where change in activity is measured by comparing activity levels before social distancing with activity levels during social distancing). Well-being was measured in terms of both positive and negative affect.

## Method

### Participants

An a priori power analysis, with power = .8 revealed a required minimum sample size of 80. Inclusion criteria for participation were: (a) engagement in some form of social distancing, and (b) being aged 18 years or over. A total of 137 participants completed the survey between May 22 and June 7, 2020. Of these, data from 42 participants were excluded because at least 10% of the data were missing, with the final sample being n = 95. Characteristics of the final sample are described in Table 1.

**Table 1:** Demographic characteristics of sample.

Variable	% of Sample
**Age**	
18–32	32
33–46	37
47–60	24
60+	7
**Gender**	
Male	26
Female	73
Other	1
**Relationship Status**	
Married/Dating	70
Single	30
**Highest Educational Level**	
High School	11
Diploma	18
Bachelor’s Degree	41
Postgraduate Degree	30
**Country of Residence**	
Australia	92.5
UK	2.2
Turkey	4.3
Canada	2.1

### Procedure

Ethics approval was obtained from the Human Research Ethics Committee, RMIT University (approval number: 22960). Consent was implied by submission of the completed anonymous survey. Written consent was not appropriate given the anonymous nature of the survey. In the unlikely event of distress as a result of participation in the study, contact details for appropriate support services were provided in the participant information statement.

Participants completed the anonymous survey online via Qualtrics. Participants reported on their current psychological state and level of engagement in activities as well as providing a retrospective account of their psychological state and level of engagement in activities one month before social distancing. For this purpose, a comprehensive catalogue of possible activities was employed. One month was selected because there was extensive discussion in the media regarding the emergence and spread of COVID-19 just before the implementation of social distancing measures which may then have already influenced participants’ psychological states. All participants completed the measures outlined below.

### Measures

#### Demographics

Participants indicated their gender, age group, highest educational level achieved, marital status, living circumstances (e.g. number of people living with them, type of dwelling), and chronic psychological or physical illness.

A scale was created for this study to measure *change in well-being*. Participants rated themselves on a series of well-being variables, (a) as they currently are, and (b) as they were one month before beginning social distancing, on a 10-point slider ranging from None (0) to Very (10). The well-being variables were: depression, anxiety, panic, loneliness, crying, cheerfulness, contentedness and laughter.

This scale allowed us to separately measure *change in positive and negative affect*. A change in negative affective state for each participant was calculated by subtracting pre-social-distancing states for depression, anxiety, loneliness, panic, and crying from during-social-distancing states for each of these variables. Positive values therefore indicate an increase in negative affect while negative values indicate a decrease in negative affect. A total change in negative affect for each participant was calculated by adding together the change scores for each of these individual variables. A change in positive affective state for each participant was calculated by subtracting pre-social distancing states for cheerfulness, contentedness, and laughter from during-social distancing states for each of these variables. Positive values therefore indicate an increase in positive affect while negative values indicate a decrease in positive affect. A total change in positive affect for each participant was calculated by adding together the change scores for each of these individual variables.

A scale was created for this study to measure *change in activity*, *modified by meaningfulness*. Participants indicated how much time they spent on various activities both (a) during social distancing (concurrently with the time of survey completion) and (b) one month before social distancing, on a slider ranging from 0 to 12 hours. Participants also indicated how important each activity is to them on a slider ranging from Not at All Important (0) to Very Important (10). (The nominated level of importance was taken to indicate how meaningful the activity is to participants.) Activities measured were: time spent outside the home, online social interaction, offline social interaction, childcare, watching television/Youtube, reading fiction, reading non-fiction, exercising, creative pursuits, listening to music, shopping, gardening or home maintenance, cleaning, playing games (online or offline), praying, meditation or yoga, social media use, employment-based work (at home or office), and doing nothing. A change in time spent on activities was calculated by subtracting pre-social distancing hours from during-social distancing hours for each activity. Positive values therefore indicate an increase in hours spent. Change in activity was multiplied by level of meaningfulness for that variable in order to achieve a measure of change in activity as a function of meaningfulness.

*The Big Five Inventory–2 Extra-Short Form (BFI-2-XS)* [21] was used to measure personality. It contains three items for each of the five subscales which measure the five personality dimensions (Extraversion, Conscientiousness, Openness to Experience, Agreeableness and Negative Emotionality). Even though the BFI-2-XS is a short form it has comparable external validity to the full BFI scale [21]. The short form maintains 71% of the internal consistency of the full scale, considered acceptable for a short scale (α values ranged from = .5 to .7) [21]. In the current study, α values ranged from .5 to .8.

The nine-item version of the *Personal Optimism and Self-Efficacy Optimism Scale* [22] was used. Personal optimism is measured using five items and self-efficacy optimism using four items, with good internal consistency for each sub-scale: α = .82 and α = .86, respectively. The scale has good convergent and concurrent validity [22]. Internal consistency was α = .87 for self-efficacy optimism and α = .73 for personal optimism for the current study.

*The Brief Resilient Coping Scale* [23] was used. This is a four-item measure of coping with stress adaptively. The scale has good convergent validity and internal consistency with α = .7 [23]. Internal consistency for the current study was α = .7.

### Statistical analysis

SPSS version 26 was used for all analyses. An inspection of the Q-Q plots and Skewness and Kurtosis metrics revealed that no variables substantially deviated from normality. Pearson correlation and linear regression analyses were undertaken to test the study hypotheses.

## Results

Other than a significant positive correlation found between change in negative and positive affect (r = .485, p = .000), there were no significant correlations found between variables. Regression analyses were nevertheless undertaken. Because regression enables one to measure the association between given predictor and criterion variables while including the effect of other predictor variables, regression analyses can be undertaken even where correlations are non-significant. No violations of assumptions were noted.

For the first regression analysis, change in negative affective state was the criterion variable. Predictors were as follows: overall change in time spent on activities, overall change in time spent on meaningful activities, age, gender, personality variables (extraversion, openness to experience, conscientiousness, neuroticism and agreeableness), level of spirituality and religiosity, self-efficacy optimism, personal optimism, and resilient coping. Using the Enter Method, it was found that the overall model was significant, F(16, 56) = 3.11, p = .003, Adjusted R^2^ = .28. Significant predictors were change in time spent on activities, change in time spent on meaningful activities, and age. Beta, t, and p values for all predictors are presented in Table 2.

**Table 2:** Beta, t and p values for predictors for regression analysis 1.

** **	**Beta**	**t**	**p**
Change in time spent on meaningful activities	-0.92	-3.76	0
Change in time spent on activities	1.08	4.7	0
**Age**	0.084	0.17	0.868
**Gender**	-1.23	0.53	0.602
***Personality***			
Extraversion	0.1	0.92	0.364
Neuroticism	0.04	0.28	0.782
Openness	-0.14	-1.1	0.277
Agreeableness	0.15	1.27	0.211
Conscientiousness	-0.24	-1.86	0.068
Religiosity	-0.07	-0.48	0.632
Spirituality	0.14	0.99	0.326
Personal Optimism	-0.04	-0.33	0.746
Self-efficacy Optimism	-0.08	-0.62	0.536
Resilient Coping	-0.1	-0.6	0.554

Note: Change in negative affective state was the criterion variable.

**Fig 1:**
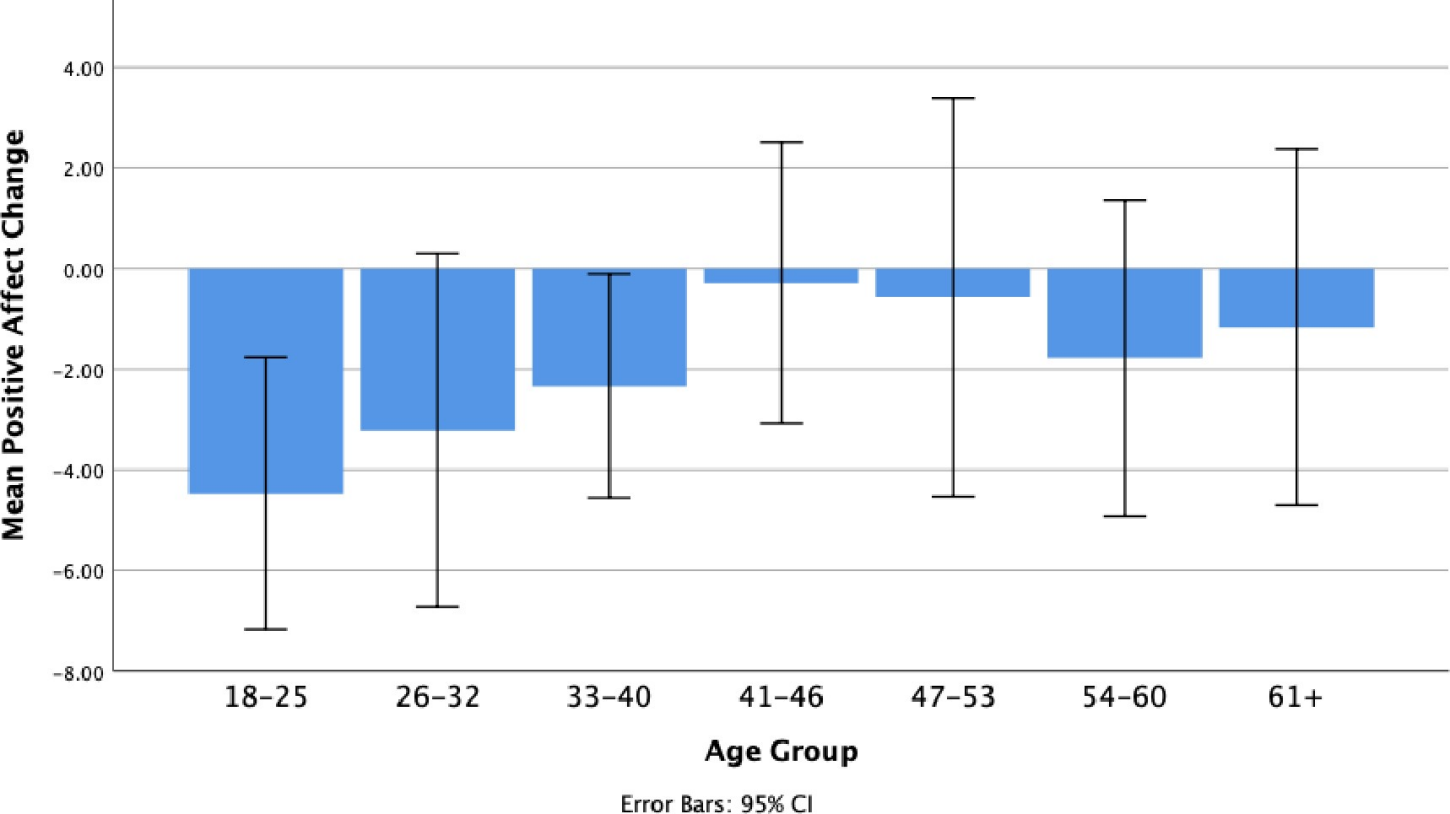
Change in positive affect as a function of age group.

For the second regression analysis, change in positive affective state was the criterion variable. Predictors were the same as for the first regression. Using the Enter Method, it was found that the overall model was significant, F(16, 56) = 2.79, p = .002, Adjusted R^2^ = .29. Significant predictors were: change in time spent on activities and change in time spent on meaningful activities. Beta, t, and p values for all predictors are presented in Table 3. A bar graph depicting changes in positive affective state as a function of age group from before lockdown to during lockdown is presented in Fig 1. As is evident, individuals in younger age groups (40 years and below) reported a greater reduction in positive affect than individuals in older age groups.

**Table 3:** Beta, t and p values for predictors for regression analysis 2.

	Beta	t	p
Change in time spent on meaningful activities	-.84	-3.47	.001
Change in time spent on activities	.84	3.61	.001
**Age**	.62	2.01	.049
**Gender**	-1.73	-1.21	.232
***Personality***			
Extraversion	-.20	-1.66	.102
Neuroticism	.22	-1.45	.153
Openness	-.08	-.63	.535
Agreeableness	-.19	-1.51	.137
Conscientiousness	-.08	-.65	.516
Religiosity	-.11	-.76	.453
Spirituality	.09	.60	.554
Personal Optimism	-.14	-1.21	.231
Self-efficacy Optimism	.03	.19	.848
Resilient Coping	-.08	-.49	.625

Note: Change in positive affective state was the criterion variable.

## Discussion

We found that increased meaningful activity predicted a reduction in both positive and negative affect. Although we predicted that negative affect would decrease with an increase in meaningful activity, we did not predict that positive affect would also decrease.

Extant studies in this area typically take psychological well-being to be a function of negative affect, thus neglecting positive affect. For instance, changes in well-being have been measured in terms of changes in negative affective states (such as depression) [15,16,18]. However, Duke et al [17] measured changes in well-being by focussing only on positive affect, thus neglecting negative affect. All of these studies nevertheless assume that a reduction in negative affect is correlated with an increase in positive affect. The possibility that positive and negative affectivity may operate independently (for instance, in our finding that meaningful activities are associated with a reduction in negative affect without a corresponding increase in positive affect) has not been anticipated or measured. However, the independent operation of positive and negative affect may be seen in a study by Stacey and Gatz [24], who found that ageing was associated with a reduction in negative affect but without a corresponding increase in positive affect.

While increased meaningful activity predicted dampened positive and negative affect, we also found that increased activity in general predicted heightened positive and negative affect. To explain this, we tentatively suggest that heightened affectivity is a homeostatic process, prompting an agent to change behavioural focus. The idea of psychological homeostasis originated in the late 1950s when this phenomenon, which had previously been applied only to physiological processes, was applied to psychological processes including drives, needs, and motivation [25]. Psychological homeostasis is now used to refer to the regulation of mood, affect and arousal. Social distancing potentially threatens homeostasis in two ways: by limiting meaningful activity and by increasing meaningless activity. We suggest that heightened affectivity functions to restore a desirable balance of activities.

Insofar as heightened affectivity involves increased positive and negative affect, it does not generate an overall reduction in activity, but rather prompts behavioural re-orientation. Heightened affectivity is therefore warranted when one needs to reduce some activities and increase others. In contrast, dampened affectivity (both positive and negative) enables an agent to maintain their behavioural orientation. Without any emotional rewards for increasing or decreasing behaviours, decreased affectivity has the effect of supporting homeostasis.

Such a homeostatic mechanism may explain why meaningful activity predicts decreased affect: it does so precisely because agents engaged in meaningful activity do not need to change their behavioural orientation. In contrast, overall busyness is associated with increased affect because busy agents are in a state of imbalance: as they are insufficiently engaged in meaningful goals, this prompts a mechanism designed to refocus their behaviour.

This particular hypothesized homeostatic mechanism facilitates the achievement of meaning in life, at the cost of sustained positive affect. This trade-off is reasonable as personally meaningful activities are not necessarily enjoyable activities; thus, a drive towards meaning in life should not prioritise positive affect per se [26]. Positive affect may be understood, not as a worthy goal in its own right, but rather as a necessary means to drive agents towards meaningful activity.

The notion that psychological well-being is associated with lowered positive and negative affect resonates with the findings of Stacey and Gatz [24]. These authors found that while older adults experienced lower positive and negative affect than a younger cohort, they also experienced greater psychological well-being than their younger counterparts. This supports our suggestion that well-being may be associated with affective stability (where the highs are not as high and the lows are not as low).

### Conclusion

We found that increased busyness during social isolation was associated with an increase in positive and negative affect, while increased meaningful activity during isolation was associated with a reduction in positive and negative affect. This suggests a homeostatic reinforcement of meaningful activity. While this reinforcement occurs at the cost of some positive affect, meaningful activity may have greater value in ultimately promoting the good life. Given that COVID-19 returns in waves, the psychological impacts of social distancing will persist over time and may indeed become accentuated with repeated iterations of social distancing. It is therefore critical to understand the factors that support well-being during social distancing.

## Limitations

Given that we asked participants to recall their behaviour and affective states approximately one month before completing the study, it is possible that errors of memory could have impacted on our findings. A diary study documenting daily behaviour and affect would be of value. Furthermore, given that we speculate that people are motivated to pursue meaningful activity rather than positive affect per se, it would be worthwhile in further research to investigate whether increasing meaningful activities enhances evaluations of life satisfaction. Based on our findings, a longitudinal study to determine changes in activity and corresponding changes in well-being would be warranted.
